# *ARHGAP4* Inhibits Proliferation and Growth of SW620 Colon Cancer Cells by Cell Cycle and Differentiation Pathways

**DOI:** 10.1155/2024/5791613

**Published:** 2024-06-06

**Authors:** Ming-Sheng Fu, Shu-Xian Pan, Xun-Quan Cai, Cui-Ting Lv, Qin-Cong Pan

**Affiliations:** ^1^Department of Gastroenterology, Shanghai Fifth People's Hospital Fudan University, No. 801, Heqing Road, Minhang District, Shanghai 200240, China; ^2^Department of Anesthesiology, Shanghai Fifth People's Hospital Fudan University, Shanghai 200240, China; ^3^Central Laboratory, Shanghai Fifth People's Hospital Fudan University, Shanghai 200240, China

## Abstract

The aim of this study is to explore the mechanism by which *ARHGAP4* regulates the proliferation and growth of colon cancer cells, and it relates to the metastasis of colorectal cancer (CRC). Various techniques including western blot, CCK8, qRT-PCR, RNA seq assay, plate cloning, subcutaneous tumorigenesis assays, and bioinformatics tools were employed to identify genes that were upregulated or downregulated upon *ARHGAP4* knockdown and their involvement in tumor cell proliferation and growth. The expression of ARHGAP4 in T and M stages of CRC uses immunohistochemistry. The expression levels of ARHGAP4 were found to be high in SW620, SW480, and HCT116 cell lines, while they were being low in HT29, LoVo, and NCM460 cell lines. Depletion of *ARHGAP4* resulted in inhibited proliferation and growth in SW620 cells and inhibited subcutaneous tumorigenesis in nude mice, whereas overexpression of *ARHGAP4* promoted proliferation and growth in HT29 cells and promoted subcutaneous tumorigenesis in nude mice. A total of 318 upregulated genes and 637 downregulated genes were identified in SW620 cells upon *ARHGAP4* knockdown. The downregulated genes were primarily associated with cell cycle pathways, while the upregulated genes were enriched in differentiation-related pathways. Notable upregulated genes involved in cell differentiation included KRT10, KRT13, KRT16, IVL, and CD24, while significant downregulation was observed in genes related to the cell cycle such as CCNA2, CDKN2C, CDKN3, CENPA, and CENPF. ARHGAP4 expression is markedly elevated in the M1 stage of CRC compared to the M0 stage, suggesting ARHGAP4 linked to the metastatic in CRC. *ARHGAP4* regulates the proliferation and growth of colon cancer cells by up- and downregulated cell cycle and differentiation-related molecules, which may be related to the metastasis of CRC.

## 1. Introduction

Colorectal cancer (CRC) remains a significant global health challenge, and CRC is the third most commonly diagnosed cancer and the second leading cause of cancer deaths in the United States [1]. In 2024, it has been estimated that 106,590 cases of colon cancer and 46,220 cases of rectal cancer will be newly diagnosed in the US, and a total of 53,010 people will die from these cancers [2]. In 2022, there were 517,106 new cases of colorectal cancer in China, and 240,010 deaths [3]. Although the prognosis of CRC has improved over the years due to advances in diagnosis and treatment options [4], however, the molecular mechanisms driving its progression and metastasis are unclear. Among the myriad of genes implicated in CRC, ARHGAP4 has emerged as a potential key player in modulating colon cancer cell proliferation and growth, with implications for metastasis. Previous studies have shown that ARHGAP4 negatively regulates the binding between GTPases and RAS family members. Rho proteins, regulated by ARHGAP4, play crucial roles in cell proliferation, differentiation, and migration [5]. These processes are implicated in various cancers, including pancreatic [6], liver [7], lung [8], and prostate cancer [9].

Our previous research found that ARHGAP4 was upregulated in CRC tissue and closely associated with poor prognosis of patients [10]. However, the specific mechanism by which *ARHGAP4* regulates the proliferation and metastasis of colon cancer cells is still uncertain. Therefore, our study aims to investigate the mechanism of *ARHGAP4* in regulating the proliferation and growth of colon cancer cells, as well as its relationship with colorectal cancer metastasis, through in vitro and in vivo assays.

## 2. Materials and Methods

### 2.1. The Expression of ARHGAP4 in T and M Stages of Colorectal Cancer

The expression of ARHGAP4 in colorectal cancer tissue by immunohistochemical follows our previous published article [10]. Calculate and compare the expression differences of ARHGAP4 in T0–T2 and T3-T4 stages of colorectal cancer, as well as the expression differences in M0 and M1 stages, and evaluation of T and M staging in CRC using the 8th edition of TNM staging standards.

The inclusion criteria for colorectal cancer samples refer to our previous published article [10].

### 2.2. Cell Culture

Normal human intestinal epithelial cells NCM460 and colon cancer cells LoVo, HT29, SW480, SW620, and HCT116 were obtained from the American Type Culture Collection (ATCC, Manassas, VA, USA). LoVo is a cell line isolated in 1971 from the large intestine of a White, 56-year-old male with a grade IV Dukes C colorectal cancer patient. HT29 is a cell line with epithelial morphology that was isolated in 1964 from a primary tumor obtained from a 44-year-old, White, female patient with colorectal adenocarcinoma. SW480 cells were isolated from the large intestine of a Dukes C colorectal cancer patient. SW620 cells are isolated from the large intestine of a 51-year-old male Dukes C colorectal cancer patient. HCT116 is a cell line exhibiting epithelial morphology that was isolated in 2018 from the rectum of a male patient with carcinoma colorectal. The cells were cultured in 6-well plates (Cellstar, Greiner Bio-one, Germany) using Roswell Park Memorial Institute (RPMI)-1640 medium supplemented with 10% fetal bovine serum (FBS), 100 U/ml penicillin, and 100 *μ*g/ml streptomycin. The cells were maintained at 37°C with 5% CO2. Passaging was performed every 3-4 days based on the growth rate. For the colon cancer cell lines, the culture medium was discarded, and the cells were washed three times with preheated PBS at 37°C. Digestion was carried out by adding pancreatic enzyme containing 0.25% EDTA to the culture dish. Under microscopic observation, the cells were considered ready for passage once they became round in shape. A mixture of 2 mL RPMI-1640 medium containing 10% FBS was added to detach the cells, which were then transferred to a centrifuge tube. After centrifugation at a low speed of 800 rpm for 5 minutes, the supernatant was discarded and the cells were washed once with PBS. Subsequently, the cells were resuspended in a fresh RPMI-1640 medium containing 10% FBS and transferred to new culture dishes for continued cultivation.

### 2.3. ARHGAP4 Transcription and Protein Level Determination

Total RNA was extracted from LoVo, HT29, SW480, SW620, and HCT116 cells using the Trizol reagent. Reverse transcription was performed using the Superscript III reverse transcriptase kit. SYBR green PCR premix was used for PCR amplification in the ABI 9700 real-time PCR system. The following primers were used: ARHGAP4-F, 5′-CTACACCTGGCCGTGTGCTC-3′, and ARHGAP4-R, 5′-CTTCCAACCGGCTCATTGTC-3′. Reference primers used were GAPDH-F, 5′-ATCCCATCACCATCTTC-3′, and GAPDH-R, 5′-AGGCTGTTGTCATACTTC-3′.

To evaluate the protein expression of ARHGAP4 in these colon cancer cell lines, protein samples were collected from the aforementioned cells and Western blot analysis was performed.

Based on the results obtained, the cell lines with the lowest ARHGAP4 expression were selected to establish stable transgenic cell lines overexpressing ARHGAP4. Similarly, the cell lines showing high ARHGAP4 expression were chosen to generate stable transgenic cell lines with ARHGAP4 knockdown.

### 2.4. Construction of ARHGAP4 Stable Transgenic Cell Line

Three pairs of shRNAs specifically targeting *ARHGAP4* mRNA were designed and cloned into the Lentivirus vector pLKO.1. Simultaneously, *ARHGAP4* cDNA was cloned into a separate Lentivirus vector, which served as the negative control. To generate a high viral titer, the vector containing the target gene and the packaging plasmid were cotransfected into HEK 293 T cells using lipofectamine 2000, following the provided instructions. After 48 hours of transfection, the virus particles present in the cell culture medium were harvested. These viral particles were then used to infect the aforementioned cell lines exhibiting low and high ARHGAP4 expression, aiming to establish stable cell lines.

### 2.5. Detection of Proliferation and Growth Ability of Colon Cancer Cells

The impact of *ARHGAP4* on the proliferation and growth potential of colon cancer cells was assessed through various experiments, including the CCK8 assay, plate cloning, soft agar cloning, and subcutaneous tumorigenesis assays. These investigations were conducted using stable cell lines that were transfected with *ARHGAP4* knockdown or overexpression. After gene knockout, further examination will be conducted to determine that there are no point mutations in the genes of the cell line.

### 2.6. The Molecular Mechanism of *ARHGAP4* Functioning in Colon Cancer

To determine the direct effect of *ARHGAP4* on gene expression, total RNA from three batches of stable transfected cell lines with *ARHGAP4* knockdown and overexpression was collected and subjected to RNA seq. A variation multiple was set based on the actual situation, and the RNA seq results were analyzed using three replicable batches as the standard. This analysis allowed us to identify genes that are positively or negatively regulated by *ARHGAP4* overexpression and knockdown, respectively. These genes are directly influenced by *ARHGAP4*. Finally, a modulation multiple was set, limiting the positive and negative regulation of *ARHGAP4* genes to 20, thus defining a set of genes regulated by *ARHGAP4*.

Using bioinformatics tools such as Gene Ontology (GO), Kyoto Encyclopedia of Genes and Genomes (KEGG), and Gene Set Enrichment Analysis (GSEA), we then analyzed which biological processes in tumor cells were involved in the positive and negative regulation of *ARHGAP4* gene sets. Representative pathways were selected for functional verification to understand the direct mechanisms through which *ARHGAP4* affects tumor occurrence and development.

### 2.7. Statistical Analysis

Data were presented as the mean ± standard error and analyzed using GraphPad Prism 8 (GraphPad Software, La Jolla, CA, USA). Each group consisted of triplicate samples, and the experiment was independently repeated three times. Statistical analysis was performed using analysis of variance (ANOVA) or Student's *t*-test, followed by unpaired comparison. A significance level of *P* < 0.05 was considered statistically significant.

## 3. Results

### 3.1. The Expression of ARHGAP4 in T and M Stages of Colorectal Cancer

**Figure 1:**
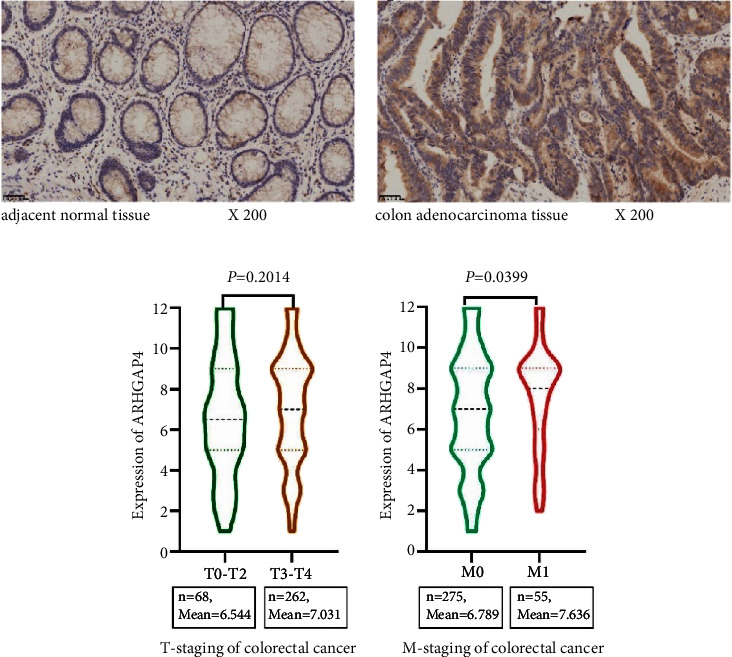
The expression of *ARHGAP4* in T and M stages of colorectal cancer. (a) *ARHGAP4* was expressed low in adjacent normal tissues of colorectal cancer and (b) was highly expressed in colorectal cancer tissues. (c) The expression of *ARHGAP4* in T0–T2 and T3-T4 stages of colorectal cancer and (d) the expression of *ARHGAP4* in M0 and M1 stages of colorectal cancer.

Immunohistochemical results showed that ARHGAP4 was lowly expressed in adjacent normal tissues of colorectal cancer (Figure 1(a)) and highly expressed in colorectal cancer tissues (Figure 1(b)). Among the 68 cases in the T0–T2 stages, the average expression value of ARHGAP4 was 6.544, while in the 262 cases in the T3-T4 stages, the average expression value of ARHGAP4 was 7.031. However, no statistically significant difference was observed between the two groups (Figure 1(c)). In the M0 stage, comprising 275 cases, the average expression value of ARHGAP4 was 6.789. Conversely, in the M1 stage, involving 55 cases, the average expression value of ARHGAP4 was 7.636, indicating a statistically significant difference between the two groups (Figure 1(d)).

### 3.2. Expression of ARHGAP4 in NCM460, LoVo, SW620, SW480, HT29, and HCT116 and Its Effects on Proliferation and Growth

**Figure 2:**
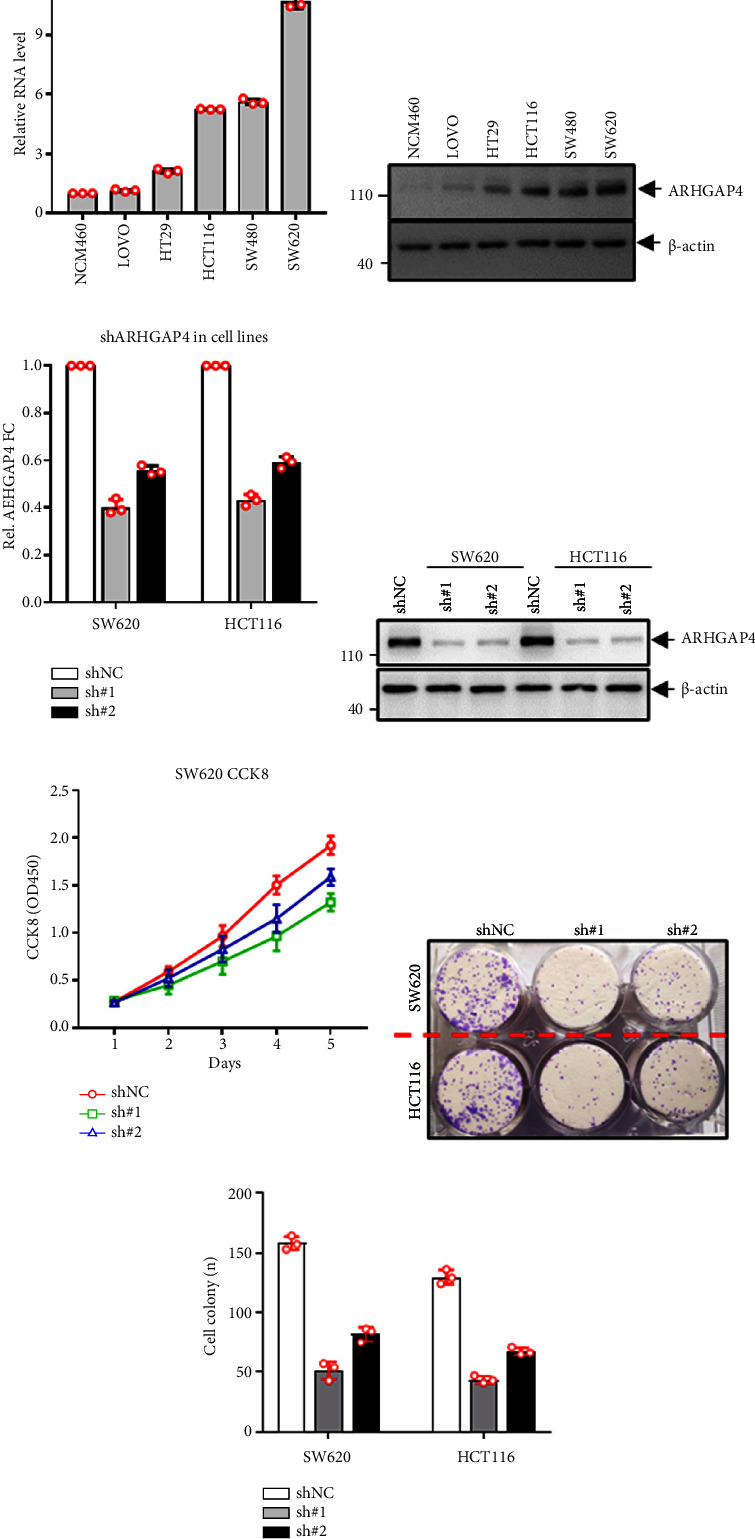
Expression of *ARHGAP4* in colon cancer cells and its effects on proliferation and growth. (a, b) *ARHGAP4* is highly expressed in malignant cancer cell lines such as SW620, HCT116, and SW480. (c, d) After knocking out sh # 1 (AGTATGAGACGCAAGTCAAAG) and sh# 2 (AGTATAACCAGAGACTCTTTG), the expression level of *ARHGAP4* was significantly reduced in SW620 and HCT116 cells, especially after knocking out sh # 1st. (e) CCK8 cell viability assay showed that knocking out sh # 1 of *ARHGAP4* significantly inhibited cell growth in SW620 cells. (f, g) Cellular colony formation assays revealed that depletion of *ARHGAP4* impaired cell proliferation and growth.

In this study, we initially assessed the mRNA and protein levels of ARHGAP4 in the normal colon epithelial cell line NCM460 and a series of colorectal adenocarcinoma cell lines. Both qRT-PCR and western blot analyses demonstrated that ARHGAP4 expression was significantly higher in malignant cancer cell lines, such as SW620, SW480, and HCT116, compared to HT29, LoVo, and NCM460 (Figures 2(a) and 2(b)). As shown in Figures 2(c) and 2(d), after knocking out sh# 1 (AGTATGAGACGCAAGTCAAAG) and sh# 2 (AGTATAACCAGAGACTCTTTG), the expression level of ARHGAP4 was significantly reduced in SW620 and HCT116 cells, especially after knocking out sh# 1. Additionally, the CCK8 cell viability assay showed that knocking out sh # 1 of ARHGAP4 significantly inhibited cell growth in SW620 cells (Figure 2(e)). The cellular colony formation assay (Figures 2(f) and 2(g)) demonstrated that depletion of ARHGAP4 significantly inhibited proliferation and growth of SW620 and HCT116 cells.

### 3.3. Overexpression of *ARHGAP4* in HT29 Cells Promoted Cell Proliferation and Growth

**Figure 3:**
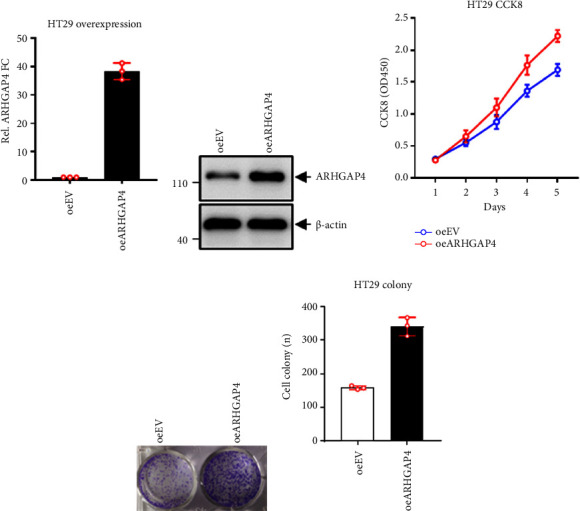
Overexpression of *ARHGAP4* in HT29 cells affects cell proliferation and growth. (a, b) After overexpression of *ARHGAP4* in HT29 cells, the expression level of *ARHGAP4* significantly increased. (c) CCK8 cell viability assay showed that overexpression of *ARHGAP4* significantly promoted the growth of HT29 cells. (d, e) Cellular colony formation assays revealed that overexpression of *ARHGAP4* promoted HT29 cells proliferation and growth.

After overexpression of *ARHGAP4* in HT29 cells, the expression level of ARHGAP4 significantly increased (Figures 3(a) and 3(b)). The CCK8 cell viability assay showed that overexpression of *ARHGAP4* significantly promoted the growth of HT29 cells (Figure 3(c)). The cellular colony formation assay (Figures 3(d) and 3(e)) demonstrated that overexpression of *ARHGAP4* significantly promoted proliferation and growth of HT29 cells.

### 3.4. Knockdown *ARHGAP4* in SW620 and Overexpression *ARHGAP4* in HT29 Affect Subcutaneous Tumor Formation Changes

**Figure 4:**
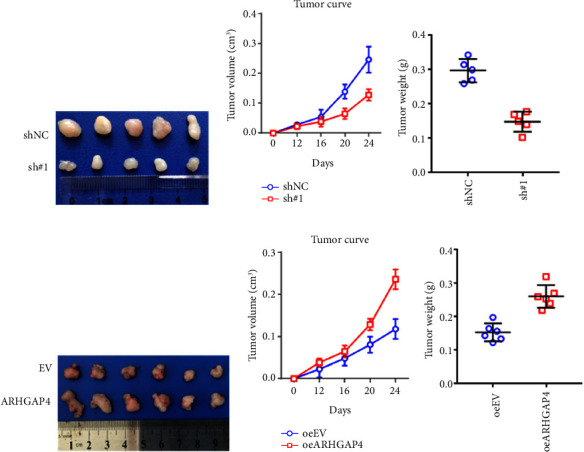
Observation of subcutaneous tumor formation changes after knockdown *ARHGAP4* in SW620 and overexpression *ARHGAP4* in HT29. (a–c) Knockdown of *ARHGAP4* in SW620 inhibits subcutaneous tumorigenesis in nude mice. (d–f) Overexpression of *ARHGAP4* in colorectal cancer cell line HT29 promotes subcutaneous tumorigenesis in nude mice.

Furthermore, we conducted subcutaneous tumor formation assays using the stable cell lines with *ARHGAP4* knockdown and overexpression. Knockdown of *ARHGAP4* in SW620 inhibits subcutaneous tumorigenesis in nude mice, as depicted in Figures 4(a)–4(c). Conversely, overexpression of *ARHGAP4* in colorectal cancer cell line HT29 promotes subcutaneous tumorigenesis in nude mice, as illustrated in Figures 4(d)–4(f).

### 3.5. Analysis of RNA Seq Results after Knocking down *ARHGAP4* in SW620 Cell

**Figure 5:**
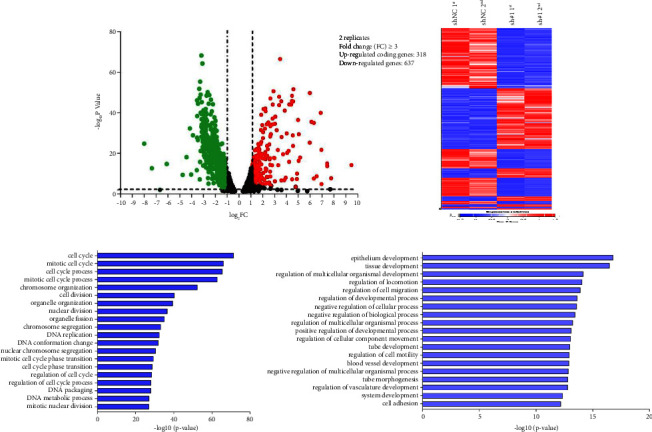
Analysis of RNA seq results after knocking down *ARHGAP4* in SW620 cells. (a) Volcano map displays that 318 genes were upregulated and 637 genes were downregulated by *ARHGAP4* knockdown. (b) The heat map shows the gene expression changes in NC cells and SW620 cells after knocking out fragments sh 1st and sh 2nd, respectively. (c) Biological processes of downregulated genes by sh#1 in SW620 cells (GO: FC ≥ 3, FDR < 0.01, top 20). (d) Biological processes of upregulated genes by sh#1 in SW620 cells (GO: FC ≥ 2, FDR < 0.01, top 20).

To elucidate the underlying mechanisms, we conducted RNA seq analysis on stably transfected SW620 cell lines. Our analysis revealed that *ARHGAP4* knockdown led to the upregulation of 318 genes and the downregulation of 637 genes (Figure 5(a)). The heat map shows the gene expression changes in NC cells and SW620 cells after knocking out fragments sh# 1 and sh# 2, respectively (Figure 5(b)). Gene ontology analysis of these significantly dysregulated genes indicated that the downregulated genes were enriched in pathways associated with the cell cycle (GO: FC ≥ 3, FDR < 0.01, top 20) (Figure 5(c)), while the upregulated genes were enriched in pathways related to cell differentiation (GO: FC ≥ 2, FDR < 0.01, top 20) (Figure 5(d)).

### 3.6. Gene Analysis of Upregulation and Downregulation after Knocking down *ARHGAP4* in SW620 Cells, as well as the Interaction between Upregulated and Downregulated Protein Molecules

**Figure 6:**
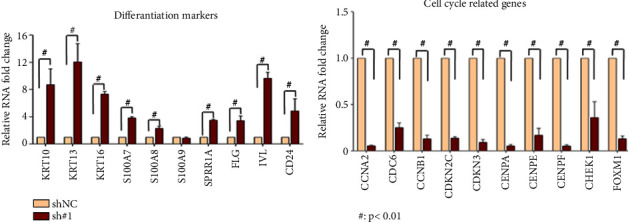
The upregulation and downregulation of gene expression after knocking out the sh # 1 gene of *ARHGAP4* in SW620 cells. (a) After knocking out *ARHGAP4* in SW620 cells, genes related to differentiation were significantly upregulated. (b) After knocking out *ARHGAP4* in SW620 cells, genes related to cell cycle were significantly downregulated.

**Figure 7:**
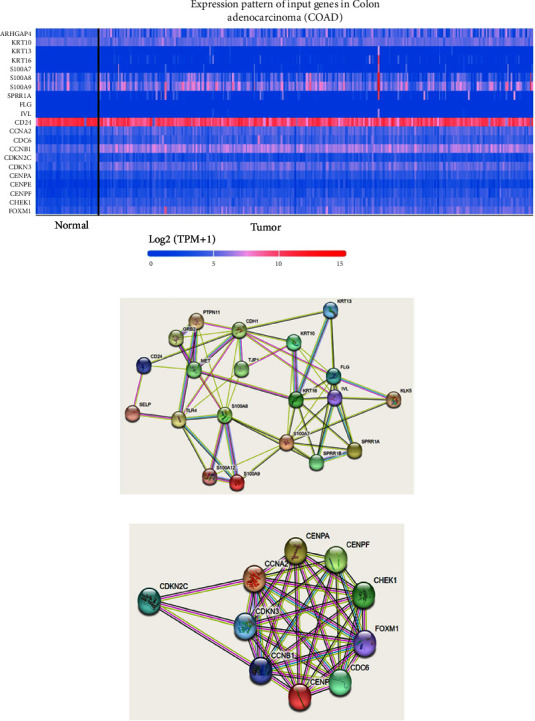
The expression of the top 10 up- and downregulated genes in colon adenocarcinoma (COAD) and the interactions between these proteins. (a) The expression of the top 10 up- and downregulated genes in COAD. (b) The interactions between protein molecules related to cell differentiation. (c) The interactions between protein molecules related to the cell cycle.

As shown in Figure 6(a), after knocking out *ARHGAP4* in SW620 cells, genes related to differentiation were significantly upregulated, while cell cycle-related genes were downregulated (Figure 6(b)). Additionally, we examined the expression levels of the top 10 up- and downregulated genes in colon adenocarcinoma (COAD) (Figure 7(a)). The interactions between protein molecules related to cell differentiation are shown in Figure 7(b) and those related to the cell cycle in Figure 7(c).

## 4. Discussion

Our previous research has indicated that ARHGAP4 is highly expressed in CRC, and this high expression is associated with a poor prognosis. ARHGAP4 has been identified as a novel prognostic marker in CRC and has shown correlations with N stage, M stage, and clinical stage [10]. Upon further analysis, it was determined that there was no significant statistical difference in the expression of ARHGAP4 between the T0–T2 and T3-T4 stages of colorectal cancer. However, a significant statistical difference was observed in the expression of ARHGAP4 between the M0 and M1 stages, suggesting that ARHGAP4 is linked to the metastatic in CRC.

The findings from this study indicate that ARHGAP4 plays a pivotal role in colon cancer cell proliferation and growth. The observed high expression of ARHGAP4 in SW620, SW480, and HCT116 cells, coupled with its low expression in HT29, LoVo, and NCM460 cells, we know that SW620, SW480, and HCT116 cells have high invasion, while HT29 and LoVo cells have low invasion, which suggests its association with the aggressive phenotype of CRC. There are functional assays, knockdown *ARHGAP4* inhibiting SW620 and HCT116 cells' proliferation and growth, and overexpression *ARHGAP4* promoting HT29 cells proliferation and growth.

Moreover, knockdown of endogenous ARHGAP4 expression in SW620 and HCT116 cells, as well as the overexpression of ARHGAP4 in HT29 cells, further validates the critical role of ARHGAP4 in CRC cell proliferation and growth. The in vivo relevance of these findings is underscored by the subcutaneous tumor formation assays, wherein ARHGAP4 knockdown impedes tumor formation in SW620 cells, while overexpression facilitates tumor formation in HT29 cells. This dual impact on in vitro and in vivo proliferation and growth highlights the potential of *ARHGAP4* as a therapeutic target in CRC. Additionally, a previous study suggests that *ARHGAP4* exerts inhibitory effects on Rho-GTPase functions, such as Rac1 and Cdc42, which are typically associated with promoting cell growth [11].

These results contribute to a deeper understanding of the molecular mechanisms underlying CRC progression and the multifaceted role of *ARHGAP4*. The observed effects on both cellular proliferation and growth and tumor formation in SW620 and HT29 cells for developing targeted therapies between *ARHGAP4* and the tumor microenvironment.

The comprehensive gene expression analysis following *ARHGAP4* knockdown in SW620 cells sheds light on the intricate molecular mechanisms underlying CRC progression. The observed changes in the expression of 318 upregulated and 637 downregulated genes provide valuable insights into the regulatory role of *ARHGAP4* in CRC cell proliferation and growth.

The downregulated genes, predominantly associated with cell cycle pathways, including CCNA2, CDKN2C, CDKN3, CENPA, and CENPF, underscore the influence of *ARHGAP4* on the control of cell division. This aligns with the functional assays demonstrating the inhibitory effect of *ARHGAP4* knockdown on CRC cell proliferation, emphasizing the pivotal role of *ARHGAP4* in cell cycle regulation. Conversely, the upregulated genes enriched in differentiation-related pathways, such as KRT10, KRT13, KRT16, IVL, and CD24, highlight the diverse effects of ARHGAP4 on tumor cell processes. The validation of these findings through qRT-PCR and further exploration of differentially expressed genes in colorectal adenocarcinoma using the UALCAN database (uab.edu) strengthen the relevance of these molecular insights to CRC.

The protein-protein interaction network analysis using the STRING online tool (STRINGdb.org) unveils key proteins, such as KRT16, S100A8, and CDKN2C, highlighting their potential roles in differentiation and cell cycle regulation. The interconnected networks formed by these proteins suggest a complex interplay influenced by ARHGAP4.

In conclusion, the gene expression profiling reveals that ARHGAP4 regulates genes associated with both cell cycle control and differentiation pathways in CRC cells. This intricate modulation of molecular processes contributes to our understanding of how *ARHGAP4* influences CRC development and progression, offering potential targets for therapeutic interventions.

Previous published studies have demonstrated that metastasizing breast cancer patients with high expression levels of KRT16 in their primary tumors experienced shorter relapse-free survival compared to those with low expression levels [12, 13]. RNA sequencing results have shown that KRT16 is overexpressed and may serve as a potential biomarker and novel therapeutic target for pancreatic cancer [14]. S100A8, also known as MRP8 or calgranulin A, is a low molecular weight calcium-binding protein belonging to the S100 calcium-binding protein family [15]. Upregulation of S100A8 expression has been observed in various tumors, including breast, pancreatic, bladder, gastric, lung, and ovarian cancers [16, 17]. Studies have indicated that S100A8 expression can influence epithelial-mesenchymal transition (EMT) and metastasis in CRC [18, 19]. Specifically, S100A8 expression was found to increase during EMT induced by TGF-*β*. Its presence in tumor cells promoted metastasis by inducing EMT, which negatively impacted patients' prognosis [20]. TCF7L2 has been shown to inhibit the cyclin-dependent kinase inhibitor C (CDKN2C)/CDKN2D, thereby restraining the growth and invasion of colon cancer cells [21]. Posttranscriptional mechanisms have been observed in hepatocellular carcinoma (HCC) where functional inactivation of the CDKN2A gene occurs [22]. *ARHGAP4* regulates the expression of KRT16, S100A8, and CDKN2C, playing a significant role in their modulation. Therefore, it can be inferred that *ARHGAP4* plays important roles in the proliferation and differentiation of colon cancer cells.

In summary, the research findings suggest that ARHGAP4 is highly expressed in CRC and is associated with a poor prognosis. It has been identified as a novel prognostic marker and has correlations with the N stage, M stage, and clinical stage of CRC. The study also reveals that *ARHGAP4* plays a pivotal role in CRC cell proliferation and growth, with its high expression being associated with the aggressive phenotype of CRC. There are unctional assays, knockdown *ARHGAP4* inhibiting SW620 and HCT116 cells' proliferation and growth, and overexpression *ARHGAP4* promoting HT29 cells proliferation and growth. The in vivo relevance of these findings is supported by subcutaneous tumor formation assays. The study further explores the molecular mechanisms underlying CRC progression and highlights the complex interplay between *ARHGAP4* and the tumor microenvironment. Gene expression analysis following *ARHGAP4* knockdown reveals changes in the expression of genes associated with cell cycle control and differentiation pathways in CRC cells. The study also identifies key proteins, such as KRT16, S100A8, and CDKN2C, which are influenced by *ARHGAP4* and may play important roles in CRC development and progression. Overall, the research provides valuable insights into the role of *ARHGAP4* in CRC and suggests potential targets for therapeutic interventions.

## Data Availability

All data are included in the article.
